# CUP-AI-Dx: A tool for inferring cancer tissue of origin and molecular subtype using RNA gene-expression data and artificial intelligence

**DOI:** 10.1016/j.ebiom.2020.103030

**Published:** 2020-10-09

**Authors:** Yue Zhao, Ziwei Pan, Sandeep Namburi, Andrew Pattison, Atara Posner, Shiva Balachander, Carolyn A. Paisie, Honey V Reddi, Jens Rueter, Anthony J Gill, Stephen Fox, Kanwal P.S. Raghav, William F Flynn, Richard W. Tothill, Sheng Li, R. Krishna Murthy Karuturi, Joshy George

**Affiliations:** a The Jackson Laboratory for Genomic Medicine, 10 Discovery Drive, Farmington, CT, USA; b The Jackson Laboratory Cancer Center, Bar Harbor, ME, USA; c Department of Genetics and Genome Sciences, University of Connecticut Health Center, Farmington, CT, USA; d Department of Computer Science and Engineering, University of Connecticut, Storrs, CT, USA; e Department of Clinical Pathology and Centre for Cancer Research, University of Melbourne, Parkville, Melbourne, Australia; f Peter MacCallum Cancer Centre, Parkville, Melbourne, Australia; g Department of Gastrointestinal Medical Oncology, Division of Cancer Medicine, The University of Texas MD Anderson Cancer Center, Houston, TX, USA; h Cancer Diagnosis and Pathology Group, Kolling Institute of Medical Research, Royal North Shore Hospital, St Leonards, New South Wales 2065 Australia; i NSW Health Pathology, Department of Anatomical Pathology, Royal North Shore Hospital, Sydney, New South Wales 2065 Australia; j Department of Anatomical Pathology, Douglass Hanly Moir Pathology, Macquarie Park, New South Wales 2113 Australia; k University of Sydney, Sydney, New South Wales 2006 Australia; l Peter MacCallum Cancer Centre, Department of Pathology, University of Melbourne, Victoria, Australia

**Keywords:** Cancer, TCGA, Classification, Machine learning, Deep learning, Cell-of-origin, Cancer-of-unknown-primary, Convolutional neural network, Inception model

## Abstract

**Background:**

Cancer of unknown primary (CUP), representing approximately 3-5% of all malignancies, is defined as metastatic cancer where a primary site of origin cannot be found despite a standard diagnostic workup. Because knowledge of a patient's primary cancer remains fundamental to their treatment, CUP patients are significantly disadvantaged and most have a poor survival outcome. Developing robust and accessible diagnostic methods for resolving cancer tissue of origin, therefore, has significant value for CUP patients.

**Methods:**

We developed an RNA-based classifier called CUP-AI-Dx that utilizes a 1D Inception convolutional neural network (1D-Inception) model to infer a tumor's primary tissue of origin. CUP-AI-Dx was trained using the transcriptional profiles of 18,217 primary tumours representing 32 cancer types from The Cancer Genome Atlas project (TCGA) and International Cancer Genome Consortium (ICGC). Gene expression data was ordered by gene chromosomal coordinates as input to the 1D-CNN model, and the model utilizes multiple convolutional kernels with different configurations simultaneously to improve generality. The model was optimized through extensive hyperparameter tuning, including different max-pooling layers and dropout settings. For 11 tumour types, we also developed a random forest model that can classify the tumour's molecular subtype according to prior TCGA studies. The optimised CUP-AI-Dx tissue of origin classifier was tested on 394 metastatic samples from 11 tumour types from TCGA and 92 formalin-fixed paraffin-embedded (FFPE) samples representing 18 cancer types from two clinical laboratories. The CUP-AI-Dx molecular subtype was also independently tested on independent ovarian and breast cancer microarray datasets

**Findings:**

CUP-AI-Dx identifies the primary site with an overall top-1-accuracy of 98.54% in cross-validation and 96.70% on a test dataset. When applied to two independent clinical-grade RNA-seq datasets generated from two different institutes from the US and Australia, our model predicted the primary site with a top-1-accuracy of 86.96% and 72.46% respectively.

**Interpretation:**

The CUP-AI-Dx predicts tumour primary site and molecular subtype with high accuracy and therefore can be used to assist the diagnostic work-up of cancers of unknown primary or uncertain origin using a common and accessible genomics platform.

**Funding:**

**NIH** R35 GM133562, NCI P30 CA034196, Victorian Cancer Agency Australia.

**Research in context**

**Evidence before this study**

Cancer of unknown primary (CUP) is defined by the presence of metastatic disease with no identified primary tumour despite extensive clinical and histopathological investigations [1]. Typically, immunohistochemical tests are performed to identify the tumour lineage targeting cytokeratins and a limited number of available cell type-specific antigens. More contemporary tests employing gene-expression, DNA methylation, and mutational profiling for the tissue of origin diagnostics have also been described, showing high performance for metastatic cancers including latent primary CUP, where a primary tumour becomes known in time. However, these tests can lack representation of important tumour types in classification and are reliant on access to specialized platforms not routinely used in diagnostic labs. Furthermore, they do not recognize the molecular subtyping of tumours that have become recognized by recent pan-cancer genomic studies that are of potential prognostic and therapeutic importance.

**Added value of this study**

In this study, we designed a deep neural network model to identify a tumour's primary site of origin using RNA-seq gene expression across 32 cancer types available data from the two largest pan-cancer genome consortia, The Cancer Genome Atlas (TCGA) and International Cancer Genome Consortium (ICGC). A machine learning classifier was also developed to discriminate the molecular subtypes of 11 cancer types once the primary site of origin is predicted. The classifier can be applied to clinical samples using widely accessible RNA-seq protocols.

**Implications of all the available evidence**

Metastatic tumours retain the gene expression profile of the primary tissue of origin enabling the development of machine learning methods for predicting tissue of origin. The development of a gene-expression tissue of origin test using the widely available RNA-seq method enables democratization of this method to other diagnostic labs. The tissue of origin classification concept can also be extended to incorporate molecular subtyping of cancer into a single test, which provides additional information to the workup of cancer patients.

Alt-text: Unlabelled box

## 1 Introduction

The vast majority of contemporary cancer treatments, including targeted therapies, are still applied with knowledge of the patient's primary tumour. However, 3-5% of all cancer patients have metastatic tumours where routine testing cannot locate the primary site resulting in a diagnosis of cancer of unknown primary (CUP) [2]. By definition, CUP's are advanced metastatic cancers and most CUP patients have a dismal prognosis with a median overall survival of 8-11 months and one-year survival of only 25% [3]. Historically, CUP patients have been treated empirically with chemotherapy which has limited benefit in most patients [4]. The paradigm of precision oncology involving detection of therapeutically actionable mutations may be of benefit for some CUP patients [5], [6], [7], [8]; however, access to these drugs can be limited, especially outside of clinical trials or through compassionate access, as with few exceptions such treatments are approved for specific tumour type indications [9,10]. The lack of knowledge of the true cancer type also puts CUP patients under severe psychological distress that may lead to clinically significant depressive symptoms [11]. Improved diagnostic methods are therefore required to improve the accuracy and speed of the diagnostic work-up of CUP tumours.

It is known that most metastatic tumours harbour a cellular phenotype that resembles their original tissue of origin. Immunohistochemistry (IHC) is commonly used in the diagnostic workup of metastatic cancers by using antibodies targeting protein antigens that have restricted cell type staining patterns. However, the utility of IHC-based classification is limited to small antibody panels and these are not standardized between laboratories. Furthermore, the application of multiple immunostains can rapidly consume often limited amounts of tumour tissue that are increasingly also required to perform other analyses, including DNA and RNA sequencing. Several studies have used molecular profiling including gene-expression (mRNA or miRNA) and DNA methylation profiling to predict CUP tissue of origin, and some of these tests have been commercialized (Table 1). Like for the detection of protein antigens by IHC, the global transcriptional or epigenetic program is retained in metastatic cancer and can, therefore, be matched to a reference of tumours of known origin using computational methods. Importantly, these molecular tests have been proven to be superior to IHC panels [[12], [13], [14]].

**Table 1:** Performance of previously published CUP classification methods.

Publication	Year	Input type	Feature number	Reported Accuracy (N)				
				Training/Cross- validation	Training tumour types	External validation	Validation tumour	Validation tumour types^+^
[87]^v2^	2011	RT-PCR assay	92	87%/85% (2206)	30	83% (187) 78% (43)	P + M	28
[15] +	2012	RT-PCR assay	92	-	-	87%^P^/82%^M^ (790^PM^)	P + M	28
[88] +	2012	RT-PCR assay	92	-	-	82.1% (184)	P + M	23
[89]^v2^	2011	Microarray	2,000	(2,136)	15	88.5% (462)	P + M	15
[14] +	2015	Microarray	2,000	-	-	89% (157)	P + M	15
[90]^v2^	2012	microRNA array	64	87% (1,282)	42	85% (509)	P + M	42
[13]	2015	Microarray	29,285	82% (450)	18	88% (94)	P + M	18
[75]	2016	DNA methylation microarray	485,577	2,790	38	94% (534)	M	21
[16]	2019	RNA-seq	17,688	97% 10,822	40 T 26 AN	86% (201)	M	40
[81]	2019	Targeted DNA sequencing	341	73.8% (7,791)	22	74.1% (11,644)	P + M	22
[82]	2020	WGS	-	91% (2,206)	24	88%^P^ 83%^M^ (2120)	P + M	16
**CUP-AI-Dx**	**2020**	**Gene Expression**	**817**	**98.54% (18,217)**	**32**	**86.96% (23)**	**M**	**6**
**CUP-AI-Dx**	**2020**	**Gene Expression**	**817**	**98.54% (18,217)**	**32**	**72.46% (69)**	**M**	**18**

v2 version 2 of CancerTypeID GEP test

+ Validation series of commercial tests

T= Tumor

AN = Adjacent normal

P= primary tumors

M= metastatic tumours.

RNA and DNA-based tissue of origin classifier methods previously reported have shown high and comparable performance. The most extensively validated RNA-based tissue of origin test is a commercial 92-gene real-time PCR based test (CancerTypeID), which reports a classification accuracy of 87% for primary and 82% in for metastatic tumours using a large validation series [15]. More recently, tests like SCOPE have leveraged TCGA RNA-seq data reporting accuracy of 89% using a neural network [16]. Another recently developed test called EPICUP uses microarray DNA methylation profiling [10]. The EPICUP study reports a classification accuracy of 94% in metastatic tumours. However, despite the utility of these tests, there are barriers to widespread clinical adoption. Tests like EPICUP rely upon access to DNA methylation platforms that are not widely accessible in diagnostic laboratories while commercial RT-PCR tests like CancerTypeID can be cost-prohibitive, which also limits their accessibility. As previously mentioned, tissue availability can be a limiting factor as genomic profiling is adopted into mainstream care. Diagnostic methods compatible with common genomics platforms such as RNA-seq can have better functionality as the raw data can also be used for other purposes, such as fusion detection.

Looking beyond tumour type prediction, molecular subtype identification—with or without the primary site identification—may also lead to enhanced therapeutic options for CUP patients. The Cancer Genome Atlas (TCGA) Research Network and International Cancer Genome Consortium (ICGC) studies have shown that cancers with a known primary site can be further classified into molecular subtypes with distinct clinical outcomes and therapeutic options [[17], [18], [19], [20], [21], [22], [23], [24]] and that shared some molecular subtypes span multiple cancers from different anatomical sites [[25], [26], [27], [28]]. For example, the mesenchymal and proliferative subtypes of ovarian cancer may be susceptible to therapies including bevacizumab and these subtypes can be used as clinical trial entry criteria [29]. Through primary site identification and then molecular subtype classification, some CUP patients may benefit from these same advances [[30], [31], [32], [33]]. However, despite the availability of genomic technologies for clinical diagnostics, identification of molecular subtypes is challenging, and due to a lack of tools and assays for pan-cancer subtyping, clinicians are often unable to utilize molecular subtype information to inform treatment decisions ([34, 35]).

**Fig. 1:**
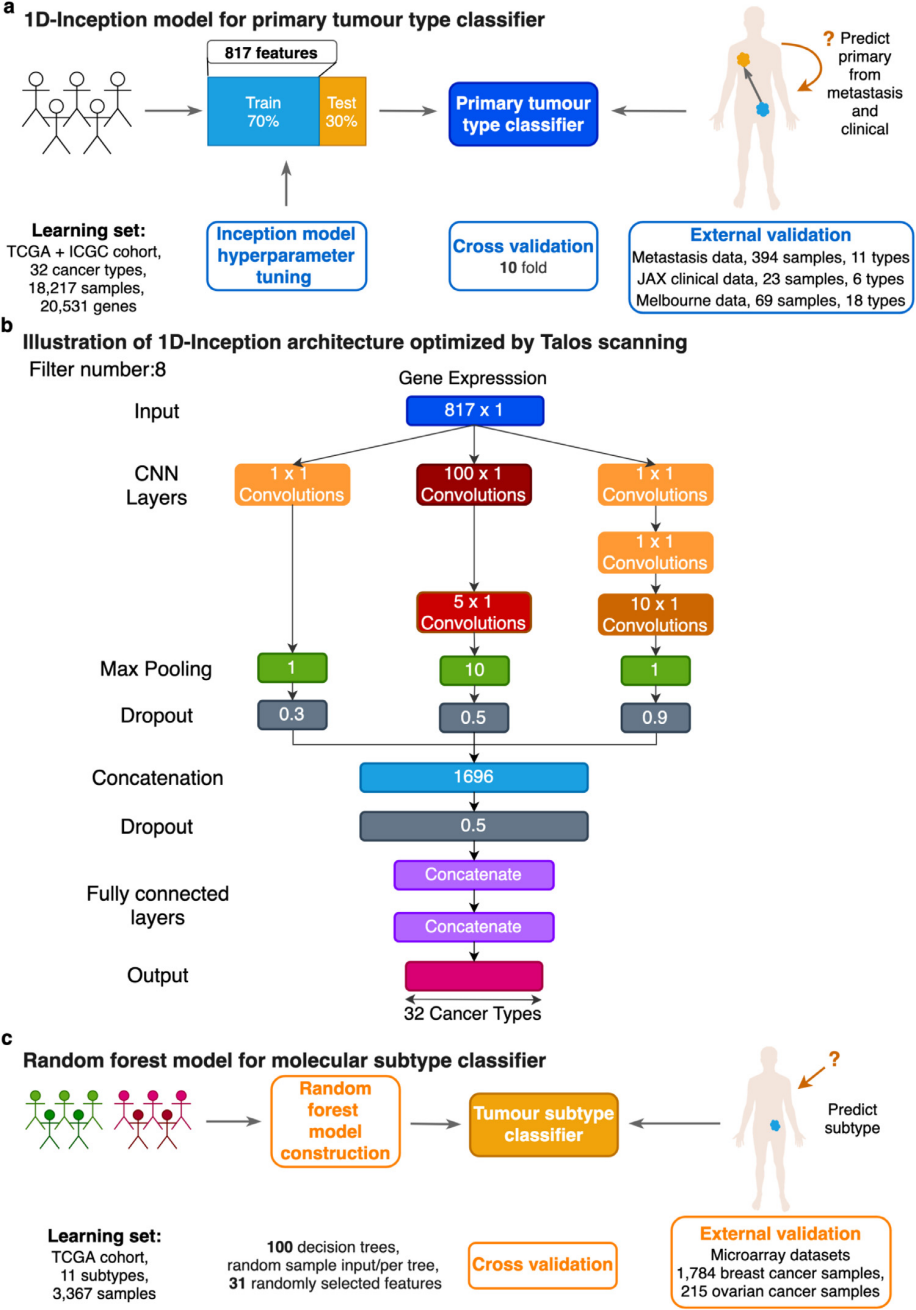
Prediction workflow for primary tumour types and subtypes. (a) Schematic showing the learning procedure used to train the 1D-Inception model from labeled TCGA and ICGC transcriptomes spanning 32 cancer types for primary tumour type prediction. Models were trained with 70% training data and validated with 30% test data on normalized and standard scaled expression profiles. 817 features were selected (see Materials and methods). Primary tumour type classification performance was evaluated via cross-validation on the learning set of TCGA and ICGC primary tumour samples and external validation utilizing primary tumour types from transcriptomes of metastatic samples and clinical samples. (b) Illustration of 1D Inception Architecture optimized by Talos [47] scanning on TCGA and ICGC dataset. Each rectangle represents a layer in the neural network. For convolutional layers, kernel size is shown, and the same kernel size layer is painted the same color. Max pooling layers are green rectangles with pooling window size inside. Dark grey rectangles are dropout layers with keep probability shown. The concatenation layer has a size of 1696 hidden nodes. This is determined by the output size from the convolutional layers. The bottom portion shows the output layer below two fully connected layers with 128 nodes individually. (c) Schematic showing the learning procedure used to train random forest (RF) models with 11 molecular subtypes for cancer subtype prediction. Models were trained and evaluated using 10-fold cross-validation on normalized and standard scaled expression profiles. N features were selected from each class (see Methods) and pooled for each fold to construct 11 molecular subtype predictors for random forest (RF). Cancer subtype classification performance was evaluated via cross-validation on the learning set and external validation utilizing breast and ovarian cancer datasets.

Here we introduce the 1D-Inception model, a machine learning framework to predict the primary site and molecular subtype of cancer samples based on the classification of gene expression data (Fig. 1). The primary classifier employs a novel type of 1D convolutional neural network (CNN) that utilizes the expression of 817 genes as input and achieves primary site classification accuracy of 96.70% when applied to an external validation set of 394 TCGA metastatic tumour expression profiles and 86.96% accuracy and 72.46% on two clinical datasets separately. Importantly, this classification accuracy is relatively robust to the absence of common IHC diagnostic markers [36,37]. In parallel, we harness random forest (RF) models to predict subtypes for the 11 TCGA cancers with established molecular subtypes, achieving a median overall accuracy of 84.06% in cross-validation. Meanwhile, the molecular subtype classifier achieved an overall accuracy of 84.18% and 79.87% in ovarian and breast external microarray datasets, respectively. Together this framework offers excellent classification accuracy to identify the primary site of metastatic cancer and allows for robust identification of its molecular subtype for the clinical management of metastatic cancers and possibly CUPs.

## 2 Materials and methods

### 2.1 Primary tumour type classification

#### 2.1.1 Training data set

TCGA gene expression data: TPM (transcripts per million) [38] normalized gene expression matrices were downloaded for each of the 33 unique cancer cohorts available from Broad Institute's Genome Data Analysis Centre (GDAC) Firehose (run 2016_01_28) [39]. We combined colon adenocarcinoma (COAD) and rectum adenocarcinoma (READ) into a single cohort (COADREAD) based on their high molecular similarities in TCGA consortium findings ([19, 40]). The gene expression matrix of each cancer was converted into a Biobase ExpressionSet object [41] for standardization, and the sets combined into a single ExpressionSet. The original expression matrix comprised 11,330 samples and 20,531 genes was reduced to 9,274 samples after extracting data for the primary tumour samples (tumour type code = “01”) and blood cancer samples (tumour type code = “03”).

ICGC gene expression data: TPM (transcripts per million) [38] normalized gene expression matrices were obtained from the International Cancer Genome Consortium (ICGC). Colon adenocarcinoma (COAD) and rectum adenocarcinoma (READ) were combined into a single cohort (COADREAD) as we described in the previous paragraph. The ICGC dataset contains 8,943 samples across 32 tumour types.

After combining TCGA and ICGC datasets, we have the gene expression data matrix with 18,217 samples spanning 32 different tumour types and 20,531 genes as the training dataset (listed in Table 2).

**Table 2:** 32 Cancer cohorts for primary classification from TCGA and ICGC.

Cohort Abbreviation	Cases	Disease Name
ACC	79	Adrenocortical carcinoma
BLCA	726	Bladder urothelial carcinoma
BRCA	2,320	Breast invasive carcinoma
CESC	568	Cervical and endocervical cancers
CHOL	36	Cholangiocarcinoma
COADREAD	873	Colon adenocarcinoma & Rectum adenocarcinoma
DLBC	48	Lymphoid Neoplasm Diffuse Large B-cell Lymphoma
ESCA	184	Esophageal carcinoma
GBM	319	Glioblastoma multiforme
HNSC	1,044	Head and Neck squamous cell carcinoma
KICH	66	Kidney Chromophobe
KIRC	1,131	Kidney renal clear cell carcinoma
KIRP	544	Kidney renal papillary cell carcinoma
LAML	346	Acute Myeloid Leukemia
LGG	969	Brain Lower Grade Glioma
LIHC	716	Liver hepatocellular carcinoma
LUAD	1,058	Lung adenocarcinoma
LUSC	974	Lung squamous cell carcinoma
MESO	87	Mesothelioma
OV	679	Ovarian serous cystadenocarcinoma
PAAD	323	Pancreatic adenocarcinoma
PCPG	179	Pheochromocytoma and Paraganglioma
PRAD	1,097	Prostate adenocarcinoma
SARC	259	Sarcoma
SKCM	537	Skin Cutaneous Melanoma
STAD	865	Stomach adenocarcinoma
TGCT	150	Testicular Germ Cell Tumors
THCA	1,067	Thyroid carcinoma
THYM	120	Thymoma
UCEC	716	Uterine Corpus Endometrial Carcinoma
UCS	57	Uterine Carcinosarcoma
UVM	80	Uveal Melanoma
**Summary**	**18,217**	

Feature gene selection on the training dataset: With the TCGA training dataset, for 1D-Inception and 1D-CNN, we selected the 40 most differentially expressed genes (DEGs) in each class (cancer type) as determined by the difference between the median expression of each gene in the in-class sample relative to the out-of-class samples (p < 0.001). Median expression was used (instead of the mean) due to its robustness to extreme values. For ResNet, we similarly selected the 70 most DEGs in each class to meet the input size requirement. DEGs may be overlapping between different classes. The DEGs sets from each class were combined, merged, and used in training each model, from which 791 and 1024 unique “feature genes” common to all external validation sets were selected for the 1D-Inception/1D-CNN and ResNet, respectively. The genes were ordered according to their chromosomal locations. In parallel, we selected 241 genes by picking the 10 most differentially expressed genes in each class (cancer type) to observe the performance of each model with a small feature set size. With the combined training dataset from TCGA and ICGC, we selected the 40 most differentially expressed genes (DEGs) in each class (cancer type) as determined by the difference between the median expression of each gene in the in-class sample relative to the out-of-class samples (p < 0.001), and combine the overlapping of different DEGs sets. Finally, 817 unique “feature” genes were selected for the 1D-Inception model construction (See Table S6 for the 817 Entrez Gene IDs).

TCGA metastatic data: To validate the primary tumour type predictor accuracy, we utilized TCGA metastatic samples (sample type code “06” (https://gdc.cancer.gov/resources-tcga-users/tcga-code-tables/sample-type-codes)) for 11 cancer types as follows (using the TCGA study abbreviations): breast invasive carcinoma (BRCA); cervical squamous cell carcinoma and endocervical adenocarcinoma (CESC); colon adenocarcinoma (COAD) and rectum adenocarcinoma (READ), which we combined into a single cohort (COADREAD); esophageal carcinoma (ESCA); head and neck squamous cell carcinoma (HNSC); pancreatic adenocarcinoma (PAAD); pheochromocytoma and paraganglioma (PCPG); prostate adenocarcinoma (PRAD); sarcoma (SARC); skin cutaneous melanoma (SKCM); and thyroid carcinoma (THCA). The metastatic gene expression matrix consisted of 394 samples spanning 11 cancer types and 16,383 genes.

### 2.2 Clinical validation data: RNA-seq data of FFPE clinical samples

Clinical validation samples included ninety-three tumours processed at two sites in the USA and Australia. Twenty-three formalin-fixed paraffin-embedded (FFPE) specimens samples representing 6 cancer types were obtained from clinical testing over 4 years in the JAX CLIA lab. Seventy metastatic FFPE tumours representing 18 cancer types were profiled at the University of Melbourne (UOM). Both sample cohorts were subject to RNA-Seq with double-blinded treatment (only tissue of origin and diagnosis were known) for clinical validation of our 1D-Inception model. All FFPE specimens were macro dissection-enriched for extraction and total RNA purified using either the Qiagen AllPrep DNA/RNA FFPE Kit or RNA FFPE Kit (Qiagen, Hilden, Germany). At JAX CLIA lab 50 ng of RNA was subjected to sequencing using the KAPA RNA PyperPrep Kit with RiboErase (HMR) protocol and sequencing by synthesis on an Illumina NextSeq 500 instrument. At the University of Melbourne Centre for Cancer Research RNA-seq libraries were prepared using the NEB-next NEBNext Ultra II Directional RNA Library Prep Kit for Illumina® and libraries sequenced on the Illumina Nova-Seq 6000. Raw BCL files generated by the sequencer were converted to FASTQ files using CASAVA. The RNA-Seq data was aligned under to the human transcriptome version hg38 using kallisto version 0.46.0 [42] running under bcbio-nextgen version1.1.6a-b'2aee4b5′ (https://bcbio-nextgen.readthedocs.io/). Raw gene expression counts were obtained from length scaled transcripts per million (TPMs) using the tximport R package version 1.12.0 [43] running under R version 3.6.0. The Ensembl [44] gene-wise annotation used by tximport was provided in the BCBio output. The distribution of the clinical datasets used for validation is shown in Table 3-4.

**Table 3:** JAX clinical dataset for external validation of primary tumour type predictor.

Cohort Abbreviation	Cases	Tumour Name
BRCA	6	Breast invasive carcinoma
COADREAD	5	Colon adenocarcinoma & Rectum adenocarcinoma
LUAD	3	Lung adenocarcinoma
LUSC	3	Lung squamous cell carcinoma
PRAD	5	Prostate adenocarcinoma
THCA	1	Thyroid carcinoma
**Summary**	**23**

**Table 4:** Melbourne dataset for external validation of primary tumour type predictor.

Cohort Abbreviation	Cases	Tumour Name
BLCA	4	Bladder urothelial carcinoma
BRCA	4	Breast invasive carcinoma
CHOL	5	Cholangiocarcinoma
COADREAD	5	Colon adenocarcinoma & Rectum adenocarcinoma
HNSC	1	Head and Neck squamous cell carcinoma
KIRC	4	Kidney renal clear cell carcinoma
LIHC	2	Liver hepatocellular carcinoma
LUAD	5	Lung adenocarcinoma
LUSC	3	Lung squamous cell carcinoma
MESO	3	Mesothelioma
OV	3	Ovarian serous cystadenocarcinoma
PAAD	5	Pancreatic adenocarcinoma
PRAD	5	Prostate adenocarcinoma
SARC	4	Sarcoma
SKCM	5	Skin Cutaneous Melanoma
STAD	3	Stomach adenocarcinoma
TGCT	4	Testicular Germ Cell Tumors
THCA	4	Thyroid carcinoma
**Summary**	**69**	

### 2.3 Normalization, filtering and preprocessing for expression data

The expression data was scaled for each patient sample independently for data normalization, i.e. the expression data were normalized by subtracting the mean and dividing by the square root of the variance of gene expression from the same patient.

All expression data were log2-transformed. After filtering, the genes in each dataset were scaled to zero mean expression and unit variance for each patient. This scaling allows expression to be measured in terms of standard deviations and affords platform-independent use of subsequently trained models.

### 2.4 Primary tumour type classifiers on TCGA dataset

To predict primary tumour types, we developed a 1D Inception model and compare it with two other deep learning models: ResNet and 1D-CNN on the TCGA dataset. Performance metrics and contingency table for all primary site predictors in cross-validation on TCGA dataset and metastasis validation are listed in Table S1-S2. The Talos hyperparameter space for each model is listed in Supplementary Text.

#### 2.5 ResNet

Due to limited samples, we chose the ResNet V50 architecture [45] implemented using Keras [46], which has the most reduced model complexity. The network input requires at least 32 × 32 2D images. Thus, we extracted the top 1024 DEGs genes following the process described in the Training section. The 1024 genes selected are ordered by chromosomal location and then reshaped to be 32 × 32 images. The output from ResNet then becomes the input into a pooling layer. In the end, it comes with a softmax output layer.

#### 2.5.1 One-dimensional convolutional neural network (1D-CNN)

1D-CNN is a good model candidate since the 1D filters learned can detect different spatial shapes in the expression matrix. Thus, we strictly ordered our 791 features according to the chromosomal location. Our experiments with 1D-CNN utilized a 1D convolutional layer followed by one max-pooling layer. Dynamic fully connected (FC) layers with ReLu activation functions were built with Talos [47]. The number of FC layers and the number of nodes were determined by nested cross-validation. Dropout filters were utilized after the max-pooling layer and the FC layers and the keep probability were selected as a hyperparameter by Talos [47]. In the end, the 32-node output layer with a softmax activation function was found optimal. The number of convolutional layer filters and the length of each filter is also selected by Talos according to the accuracy of the training validation set.

The 1D-CNN model was constructed using the Keras framework (v2.2.4) [46]. The hyperparameters selected by Talos [47] were as follows: batch size of 32; optimal performance achieved by having no dropout; 32 filters, each with a length of 4; four hidden layers with 64 nodes in each layer; the learning rate used for training is 0.02; the number of epochs of 200; and weight initialization using the Xavier normal method [48].

#### 2.5.2 1D Inception network with optimized hyperparameter setting by Talos (1D-Inception)

**Fig. 2:**
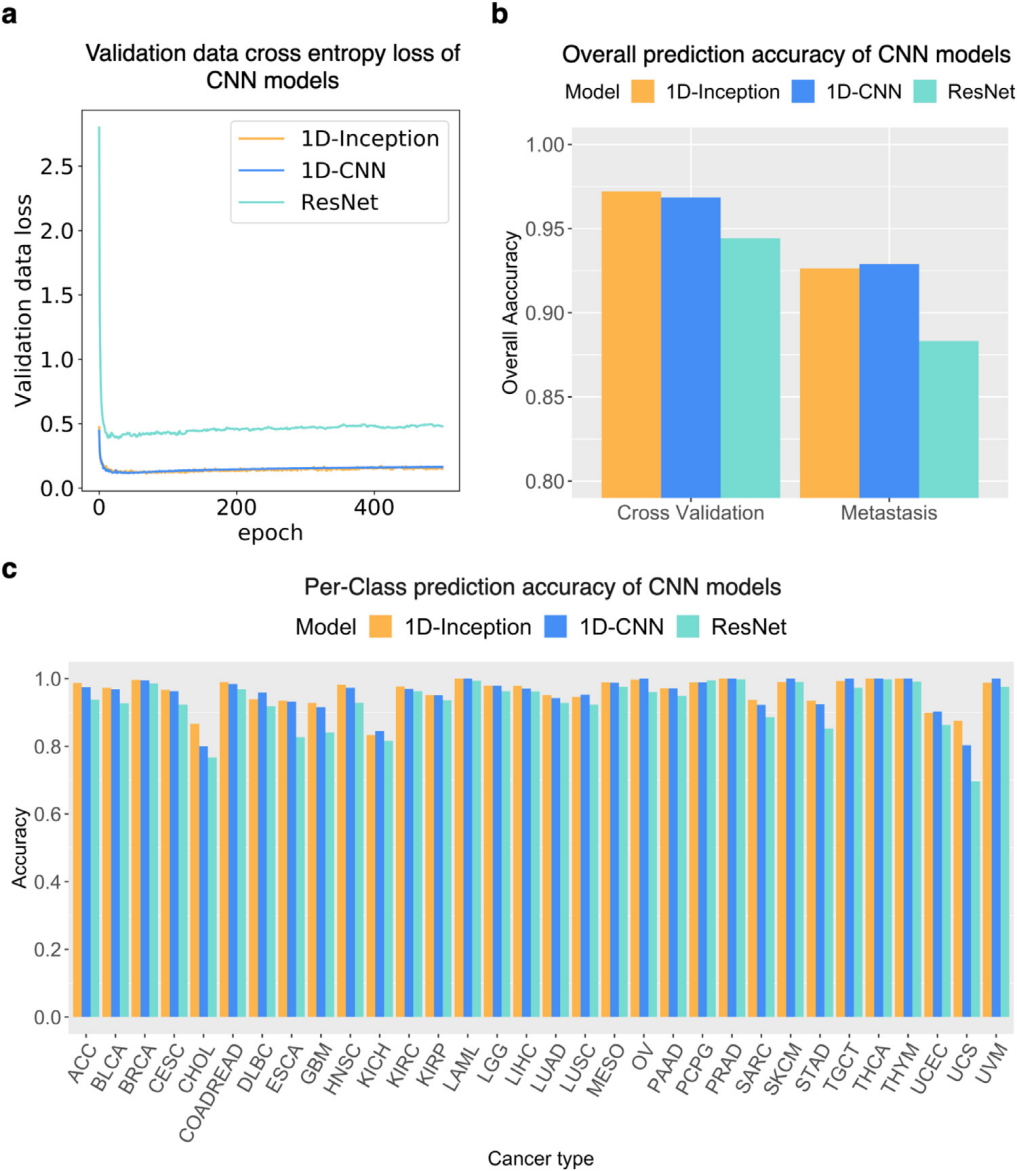
Primary tumour type prediction performance of CNN models on the TCGA dataset. (a) Validation data cross-entropy loss of CNN models. One can observe that the training processes of all three models successfully converged. (b) Overall prediction accuracy of CNN models in cross-validation and external metastasis validation. (c) Per-class accuracy performance of CNN models.

The 1D-Inception network is used to enhance the 1D-CNN network by considering multiple 1D convolutional kernels with different sizes at the same time. We chose this architecture because the 1D-Inception model was found to have superior performance on image data sets [49]. The filter size and number are also tuned as hyperparameters. The advantages of combining different size kernels can be confirmed by the optimal architecture picked by Talos [47], as shown in Fig. 2c, where the combination of different kernel sizes gives better performance than the other settings.

### 2.6 Cross and external validation procedure

The schema for predictor design for primary site classification is depicted in Fig. 1a.

All CNN models, 1D-Inception, 1D-CNN, and ResNet were trained using the same feature selection and cross-validation schedule on the TCGA dataset. Each model was then trained using a 10-fold cross-validation procedure as follows. The expression set was partitioned into 10 random subsamples and for each partition: [1] the selected subsample was used as the testing set and the remaining 9 were combined into a training set; The training comprised 500 epochs, using the Adam [50] optimizer with 32 as the batch size and 0.001 as the learning rate [2] the model was trained using the selected 791 gene features (1024 for ResNet); and [3] predictions for the selected partition were recorded. The cross-validation procedure yields an estimate of model performance using the selected parameters.

### 2.7 1D-Inception model on TCGA and ICGC dataset

The 1D-Inception architecture for TCGA and ICGC dataset is shown in Fig. 1b. Here each convolutional module has 1, 2, or 3 layers, respectively. The filter size and number are also tuned as hyperparameters and the Talos hyperparameter space is listed in Supplementary Text.

Because this network allows the three convolutional modules to have different max-pooling layer window sizes and dropout layer keep-probabilities, we can utilize as few as 817 genes in the model to achieve a high overall top-1-accuracy of 98.54% in cross-validation and overall top-5-accuracy of 99.94%. This outperforms alternative methods that usually consider the whole gene set. This is because the lower-dimensional data has fewer patterns than the high dimensional data, thus it requires more kernel size options to detect the limited number of patterns. A third strength of this architecture is that the redundant layers can be reduced through Talos performance checking. As shown in Fig. 1b, some CNN layers have filters of size 1 × 1, indicating that projecting the output from previous layers directly can benefit performance. Similarly, some max-pooling layer window sizes are optimized to be 1, which means that no max-pooling layer can provide better performance.

## 3 Molecular subtype classification

### 3.1 Training data set: Molecular subtype from TCGA gene expression data

Molecular subtype information was downloaded from cBioPortal [51,52] for 3,367 samples derived from TCGA dataset from the following primary tumours: glioblastoma multiforme (GBM), stomach adenocarcinoma (STAD), breast (BRCA), ovarian (OV), prostate (PRAD), and lung squamous cell cancers (LUSC). Further annotations were curated from the following supplemental data files: lower-grade glioma (LGG) [53], head and neck squamous cell carcinoma (HNSC) [18], uterine corpus endometrial carcinoma (UCEC) [54], cutaneous melanoma (SKCM) [55], kidney renal papillary cell carcinoma (KIRP) [56] and kidney clear cell carcinoma (KIRC) [57], and lung adenocarcinoma (LUAD) [23]. R (version 3+) scripts were written to extract relevant information (e.g. sample ID, specific subtype) from downloaded data and supplemental files. These scripts are available in the public project GitHub repository as described under Code availability.

For each of the 11 primary tumour types with established molecular subtypes, a model was constructed as described above using the scaled, log2-transformed expression of the sample corresponding to the selected primary type as input. For each cancer type, features were selected by computing the differential gene expression (p<0.001) in each subtype in comparison with the other subtypes of the same cancer type. The list of gene features is shown in Table S7.

### 3.2. External validation dataset: public microarray data

For the external validation of our subtype predictors, we acquired two additional microarray datasets. The first, accession number GSE9899, contains 215 ovarian cancer samples [58] and the second, EGA study EGAS00000000083 (https://www.ebi.ac.uk/ega), contains 1,784 breast cancer samples [59]. Both datasets comprise four molecular subtypes each: mesenchymal, immunoreactive, differentiated, and proliferative for the ovarian set; and basal-like, HER2-enriched, luminal A, and luminal B for the breast set.

### 3.3 Machine learning algorithms for subtype classification

We evaluated several popular machine learning algorithms to develop predictors for molecular subtype identification and chose random forest (RF) model using R-packages *randomForest* (version 4.6-14) for the training and testing and *caret* (version 6.0-79) for tool development. Unless otherwise specified, default parameters were chosen for model construction. Performance metrics for subtype predictor and pan-cancer predictor are listed in Table S3-S5. Comparable results were obtained using XGBoost, another ensemble model (Supplementary tables S9) but Random Forest is chosen as the default subtyping algorithm because of the superior performance in the external validation set.

#### 3.3.1 Random forest

The random forest algorithm employs a collection of decision trees constructed from bootstrapped input data and classification is done by majority voting among the ensemble of trees [60]. As single decision trees are prone to overfitting, we used multiple trees constructed from randomly sampled copies of the input data to enable the consensus classification to be robust and extensible to new samples. Each of our random forest models constructed 1000 trees, each constructed from randomly sampled input with replacement, and each decision tree node used 31 randomly selected features to partition the tree.

### 3.4 Ethics statement

The Jackson Laboratory (JAX) Institutional Review Board (IRB) has reviewed the

Determination of Human Subjects Research form for the project indicated above and has determined that this project does not meet the definition of human subjects research under Laboratory policy and applicable Federal Regulations.

This determination is based on the fact that the genomic summary data from RNA-seq assays is provided with no individual-level genomic data shared in publication. RNA-seq analysis of patient samples at the University of Melbourne was done under an approved protocol by the human research ethics committee at the Peter MacCallum Cancer Centre (Protocol: 11/117).

### 3.5 Statistical analysis

Each classification algorithm (predictor) was compared using overall accuracy, per class accuracy, sensitivity, and specificity. For datasets with class-imbalance, reporting accuracy alone is a misleading metric to gauge performance. For example, more than 50% of the training data from the TCGA network consists of tumours from only 9 of 32 classes, with BRCA making up 12% of the training dataset, and tumour classes are similarly imbalanced in the external validation datasets. Instead, we report classification performance as a combination of overall and per-class performance. Per class metrics are computed using a one-versus-all scheme. TP = true positive, TN = true negative, FP = false positive, FN = false negative: Metrics of performance are calculated as follows:

$$Overall\;accuracy=\frac{total\;number\;of\;correct\;tumor\;type\;predictions\;by\;classifier\;}{the\;total\;number\;of\;tumor\;samples}$$

Per class:

$$Per\;class\;accuracy=\frac{Number\;of\;tumor\;samples\;precited\;as\;type\;A\;by\;classifier}{Tumor\;sample\;numbers\;in\;the\;specific\;type\;A}$$

$$Precision=\frac{TP}{TP+FP}$$

$$Sensitivity\;\left(Recall\right)=\frac{TP}{TP+FN}$$

$$Specificity=\frac{TN}{TN+FP}$$

$$F1=\frac{2*precision*recall}{precision+recall}$$

Additional metrics such as specificity, sensitivity (recall), and F1 score are included in supplementary tables S1-S5, S8.

## 4 Results

### 4.1 Precise classification of primary tumour types across platforms

RNA-seq cancer data was sourced from the TCGA and ICGC compendium datasets. To generate a classification training dataset, we combined COAD and READ into one single cohort (COADREAD) based on TCGA consortium findings [40] and kept only primary tumour samples and blood cancer samples for further analysis (see **Methods**); it is important to note that the training data contained no metastatic tumour samples. The filtered cancer cohorts (18,217 samples, 32 cancer types) are shown in Table 2. We harnessed the 1D-Inception architecture neural network, a novel CNN model for primary tumour type prediction (Fig. 1a). The Inception model utilizes multiple one-dimensional convolutional layers with different kernel sizes to operate on a gene expression input vector, as presented in Fig. 1b and described in detail in the Methods.

To gauge the performance of our 1D-Inception model, we first compared its performance to that of two other popular CNN models, the 1D convolutional neural network (1D-CNN) model and Residual Net (ResNet) [61] using the TCGA dataset only. The primary tumour type prediction performance of the three CNN models applied to the TCGA dataset is shown in Fig. 2 and Figure S1. Fig. 2a illustrates the training process by plotting the validation set cross-entropy loss on model training epochs and shows that the training for all three models converged without inducing overfitting. The 1D-Inception model achieved the best 10-fold cross-validation performance (Fig. 2b) among the three CNN models. The overall top-1 cross-validation accuracy was 97.20%, 96.85%, 94.43% for 1-D Inception, 1D-CNN, and ResNet, respectively (Fig. 2b), and the overall top-5 cross-validation accuracy was 99.85%, 99.77%, 99.06% respectively. The 1D-Inception model mean sensitivity for cross-validation is 0.9580 while the mean precision is 0.9607. The per-class prediction accuracy (positive predictive value) (Fig. 2c) and per-class F1 score (Figure S1c) also revealed the 1D-Inception model's excellent performance in cross-validation. The cross-validation confusion matrices of the 1D-Inception model, the 1D-CNN, and ResNet model in the 10-fold cross-validation experiment with the TCGA dataset are shown in Figure S2. In summary, our 1D-Inception model performed the best among the three CNN models.

**Fig. 3:**
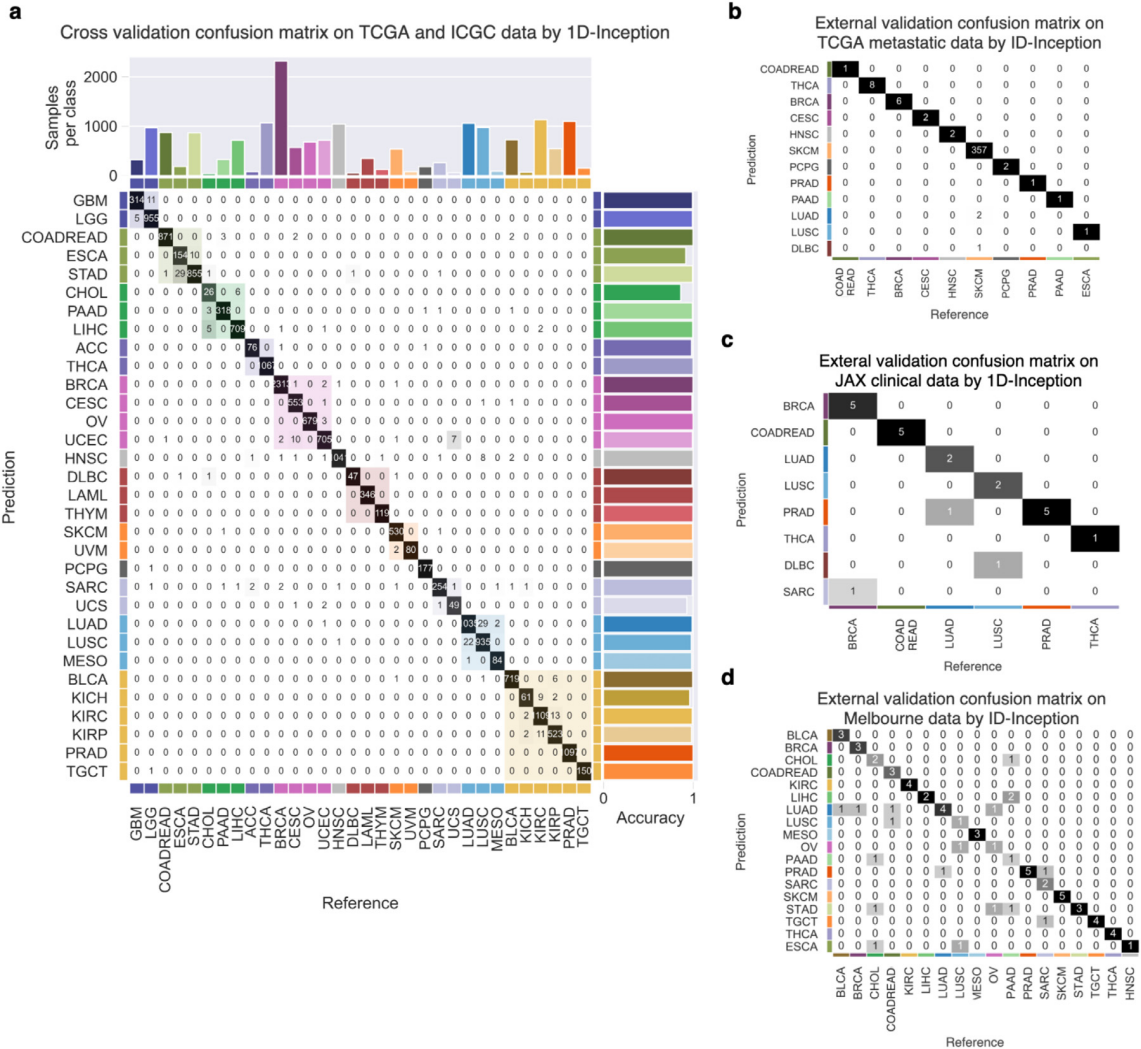
Cross- and external validation of primary tumour type predictor. The 1D-Inception model was constructed for primary tumour type prediction. 32 primary tumour types are grouped by the pan-organ system. (a) Inception model confusion matrix for cross-validation of 32 primary tumour types on TCGA and ICGC dataset. Accuracy for each prediction class is shown to the right of the table. (b) 394 expression profiles of TCGA metastatic tumours from the primary site of origin spanning 11 organs were classified by the primary tumour type predictor. (c) 23 expression profiles of clinical datasets spanning 6 cancer types were classified by primary tumour type predictor. (d) 69 expression profiles of Melbourne dataset spanning 18 cancer types were classified by primary tumour type predictor. Text in contingency table cell *c_j,i_* of (b), and (c) shows the number of class *i* tumour samples classified as class *j*. The heatmap of the confusion matrix is coloured in grayscale. Colour shading along with the main diagonal shows pan-organ groups.

To further improve our 1D-Inception performance, we combine the TCGA and ICGC datasets as a combined dataset to redo the training process and updated the architecture (Fig. 1b). The 1D-Inception model confusion matrix on TCGA and ICGC datasets is shown in Fig. 3a, achieving 98.54% accuracy in the 10-fold cross-validation experiment. It is important to note that every tumour sample was classified by our model; no samples were excluded from classification, either by a sample quality metric or through a lack of consensus during label assignment.

The major misclassifications observed during cross-validation were primarily within organ systems (Fig. 3a). Of note was the misclassification of uterine carcinosarcoma (UCS) as uterine corpus endometrial carcinoma (UCEC). Histologically, USC presents features of both UCEC and sarcoma (SARC) [62] and it is now accepted that the sarcomatous component of carcinosarcoma is derived from the carcinoma as a result of transdifferentiation (epithelial–mesenchymal transition) during tumour evolution [62], [63], [64], [65]. Similarly it would be expected that the distinction between esophageal adenocarcinomas (ESCA) and stomach adenocarcinoma (STAD) would be challenging, given that ESCAs originating near the gastroesophageal junction (GEJ) are STAD-like with the distinction in clinical practice normally made primarily based on anatomical location of the primary tumour rather than on any morpholgicaly, immunohistochemical or biological difference [66], and gastroesophageal carcinoma showing a progressive gradation of subtypes [67].

**Fig. 4:**
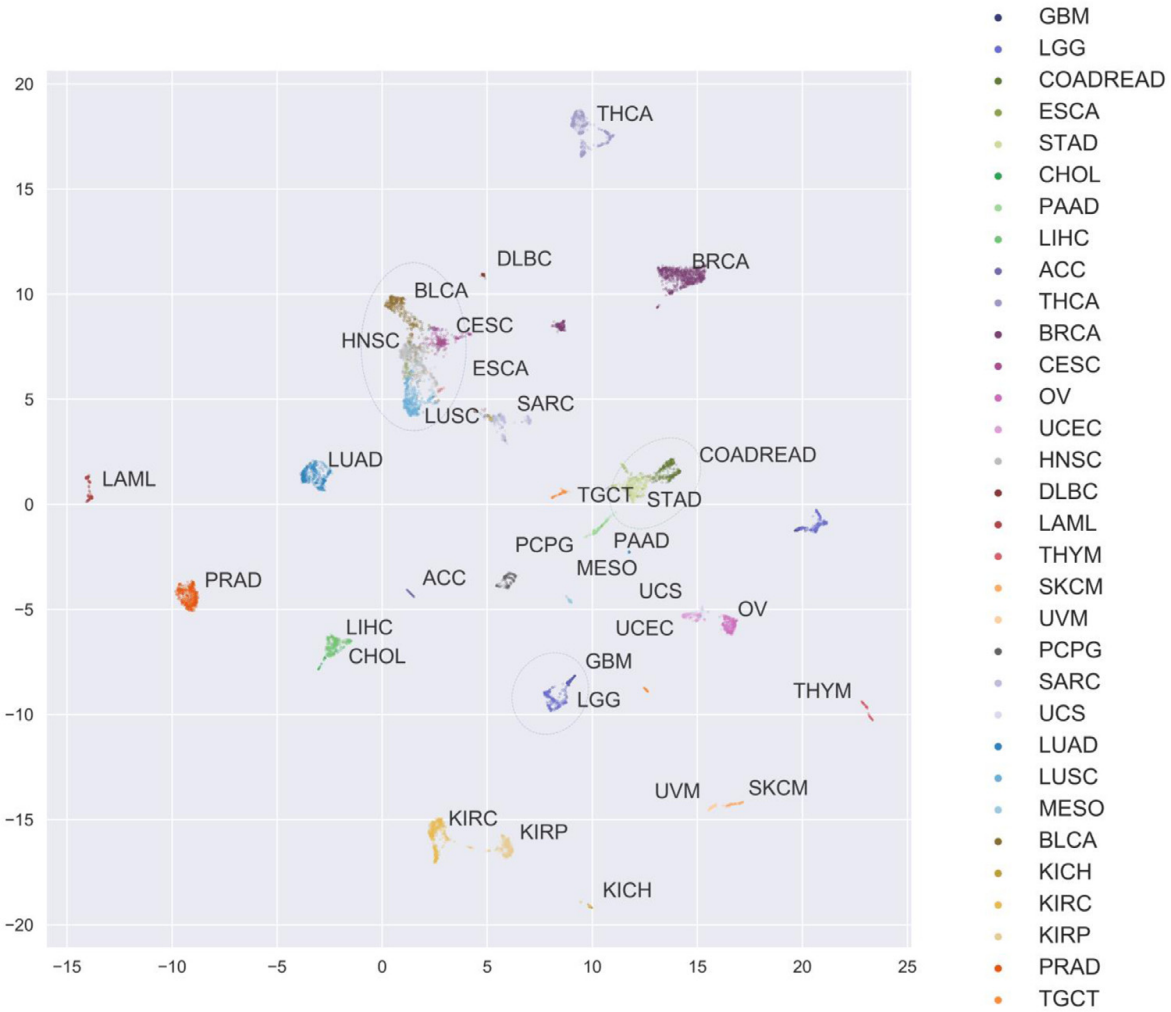
Unsupervised embedding of expression profiles reveals relationships among primary sites. Expression profiles from all samples in the TCGA dataset were embedded into two dimensions using uniform manifold approximation and projection (UMAP) [86] and colored by primary tumour type. For each cancer, labels are placed near the centroid of the expression profile in the UMAP latent space. Anatomical and histological relationships are emergent and add context to the most common misclassifications in Figure S2a. The following groups of cancers are highlighted with green, blue, and purple ellipses, respectively: i) COADREAD, STAD; ii) BLCA, CESC, ESCA, HNSC, LUSC; iii) GBM, LGG.

To understand these misclassifications, the expression profiles of every training sample were embedded into a two-dimensional latent space using UMAP (see **Methods**) and coloured by primary tumour type from the TCGA dataset (Fig. 4). Several anatomical and histological structures emerged from the embedding. Some cancers were observed to form disparate, well-separated clusters by organ systems, such as the brain (GBM-LGG), liver (LIHC), gallbladder (CHOL), and kidney (KIRC, KIRP, KIRH). Other cancers were clustered in accord with histological features, such as melanomas (SKCM, UVM) and squamous cell cancers (BLCA, CECS, HNSC, LUSC, and some ESCA), consistent with recent reports that these cancers share phenotypes and subtypes [25,26,28]). The core gastrointestinal tract cancers cluster tightly, with COAD and READ embedded in one mass, reaffirming their treatment as a single cohort, COADREAD, which is adjoined by STAD and some ESCA samples. ESCA samples segregated into two clusters, consistent with both esophageal adenocarcinoma (clustered with STAD) and squamous cell carcinoma (clustered with LUSC, HNSC, etc.) when classified under ESCA [68]. Similarly, the known similarities between USC, UCEC, and SARC emerged clearly, with the embedding of USC forming a bridge between UCEC and SARC clusters. We also observed two distinct clusters of SARC samples, one most similar to USC and the other most similar to UCEC. As this embedding is heavily dependent on the input samples and number thereof, it may be that some misclassifications are unavoidable without a larger cohort of samples.

### 4.2 Application of CUP-AI-Dx to metastatic cancers

As stated above, the identification of the primary site from a metastatic sample is a significant clinical challenge. We assume that metastases, including CUPs, should be expected to retain at least a partial transcriptional signature of the primary site. Therefore, we used our classification model to predict the primary site of 394 metastatic samples across 11 known primary sites from the TCGA dataset. We first trained our model on the TCGA dataset and then compared the performance with 1D-CNN and ResNet. Each deep-learning model achieved overall top-1-accuracy 92.64%, 92.89%, 88.32% for 1D-Inception, 1D-CNN, and ResNet, respectively (Fig. 2b), and overall top-5-accuracy of 97.72%, 97.72%, 96.19%, respectively. The 1D-Inception model had better external validation accuracy (91.87%) with smaller feature sizes (241 genes) compared to 1D-CNN (87.56%) (Fig. S3).

After combining the TCGA and ICGC datasets to train the 1D-Inception network, performance is further improved to top-1-accuracy at 96.70% in TCGA metastatic samples and the confusion matrix is shown in Fig. 3b. Notably, this performance is achieved by only considering the expression of 817 genes. This validation of our 1D-Inception model using TCGA metastatic samples gives strong support to the hypothesis that metastatic samples retain the molecular profile of the primary tumour and can, therefore, be used to predict the primary site of the tumour.

To extend our tool into clinical application, we generated two clinical datasets, JAX clinical dataset with 23 FFPE samples across 6 cancer types (Table 3) and Melbourne dataset with 69 FFPE samples across 18 cancer types (Table 4), as independent datasets for external validation. Our 1D-Inception model trained on TCGA and ICGC dataset achieved overall accuracy at 86.96% in the JAX clinical dataset (Fig. 3c) and 72.46% in Melbourne dataset (Fig. 3d). For this external validation, the mean sensitivity and precision are 0.8611 and 0.8095, respectively. The performance on the clinical dataset demonstrates that our 1D-Inception model can be applied in a clinical setting for single sample classification.

To show our model's robustness, we firstly remove 23 genes from the IHC diagnostic marker genes [36,37] in the metastatic dataset, finding that 1D-Inception performance is not affected in the absence of IHC marker key genes (Figure S4a). Furthermore, we randomly remove k(= 5, 10, 20, 50, 200) feature genes and show that prediction accuracy is only slightly affected when some of the features genes are not present (Figure S4b). It is important to reiterate that our 1D-Inception classifier can identify the tissue of origin with only 817 feature genes as input and is robust to the absence or presence of key diagnostic genes used clinically. Such a model may enable a more cost-effective and precise diagnosis of CUPs.

### 4.3 Subtype specific classification accurately identifies molecular and pan-cancer subtypes

Molecular subtypes have been defined for 11 cancer types: BRCA, HNSC, KIRC, KIRP, LGG, LUAD, LUSC, OV, PRAD, SKCM, and STAD. Each of these primary types has two to four molecular subtypes. For example, breast cancers are frequently subtyped into Basal-like, Her2-enriched, Luminal A and Luminal B. Such subtyping is growing in clinical relevance and can be used as a predictive marker for therapeutic approaches [69]. However, there are relatively few available datasets with identified molecular subtypes. Our deep learning framework requires relatively large training datasets to perform well, so we chose to build random forest (RF) models for molecular subtype identification.

**Fig. 5:**
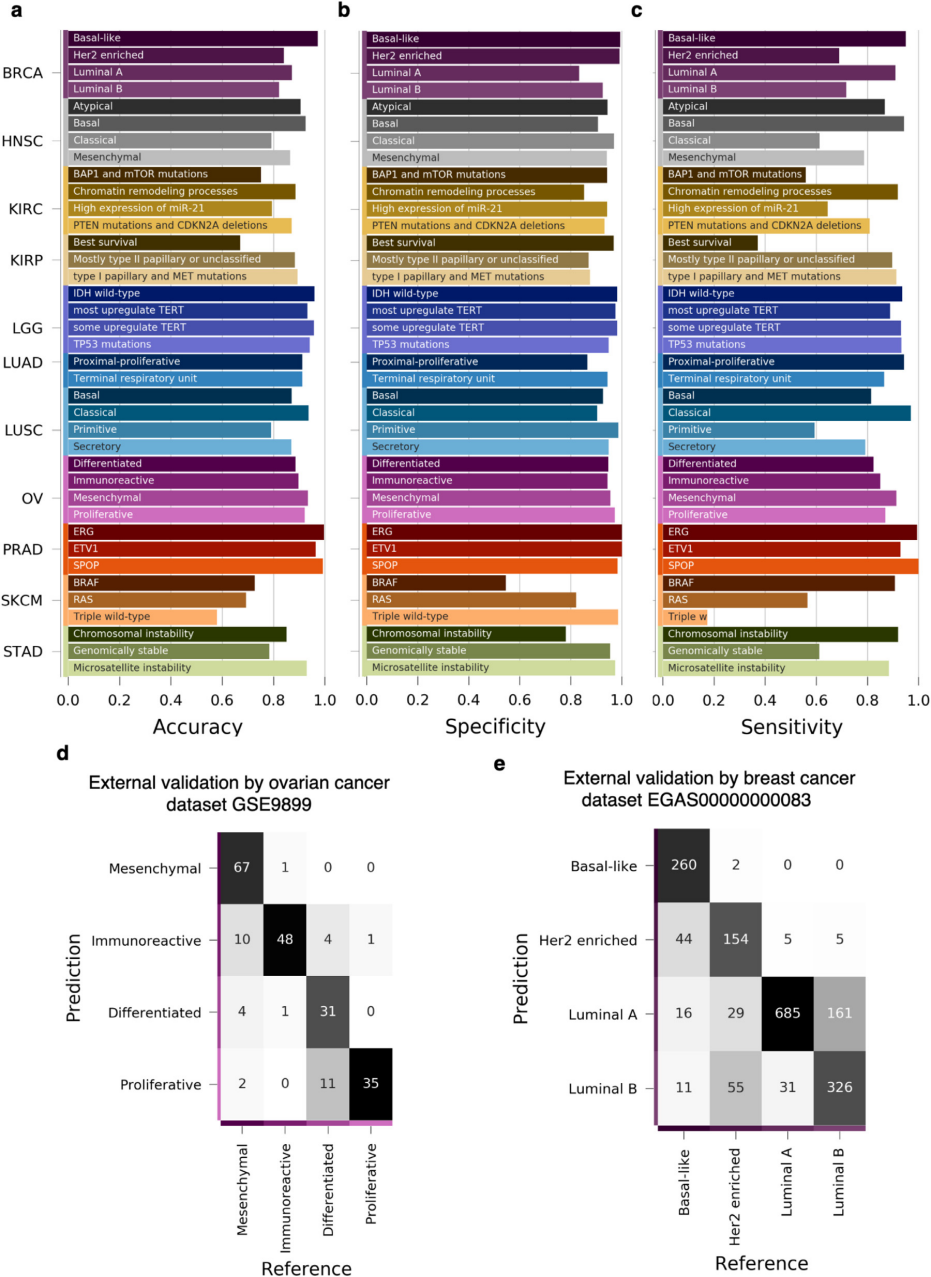
Cross- and external validation of molecular subtype predictors. A predictor of molecular subtypes was constructed for each of 11 primary tumour types, spanning 38 molecular subtypes on the TCGA dataset. (a) Per-class accuracy, (b) specificity, and (c) sensitivity of molecular subtype classifications evaluated through cross-validation (Fig. 1c). To further validate these subtype predictors, ovarian (d) and breast (e) subtype predictors were used to predict the respective molecular subtypes in two external datasets (GSE9899 and EGAS00000000083, respectively).

Eleven models were constructed, one model for each primary tumour type, into its molecular subtypes, as illustrated schematically in Fig. 1b. The accuracy (positive predictive value), specificity, and sensitivity per subtype are shown in Fig. 5a-5c. The best performing subtype predictors, LGG, LUAD, PRAD, had median sensitivity above 90%, with PRAD yielding nearly perfect classification.

Recent studies by The Cancer Research Network have examined multi-omics data for several groups of related cancers and have identified shared molecular subtypes in each pan-cancer group [[25], [26], [27], [28]]. For example, Liu et al. show that there are five distinct molecular subtypes shared among hypermutated ESCA, STAD, COAD, and READ tumours. These studies have identified 5 pan-gynecological, 6 pan-squamous-cell, 5 pan-gastrointestinal, and 6 pan-kidney cancer molecular subtypes, respectively.

These pan-cancer molecular subtypes provide a separate opportunity to test our methodology as these subtypes are defined wholly or in part by multiple types of genomic information other than gene expression data. In addition to building classifiers for molecular subtypes of individual primary tumour types, we constructed subtype classifiers for each pan-cancer group above. Cross-validation performance metrics for the four classifiers built from these samples are listed in Table S5. Despite the definition of many of these pan-cancer subtypes consisting of multi-omics signatures, we found moderate performance among all four expression-based classifiers, with the pan-squamous-cell classifier with the highest performance (83.5% overall accuracy and 88.2% median sensitivity) and pan-gynecological performing the worst with 60.4% accuracy and 62.4% median sensitivity. The pan-gynecological cluster with the worst performance, subtype C2 with only 41% sensitivity, was almost solely differentiated from subtype C1 by hypermutation patterns [25].

### 4.4 Subtype predictors are accurate on external data of different platforms

To further validate the cancer subtype predictors, we classified samples from two external datasets: ovarian cancer (GSE9899) [58] and breast cancer (EGAS00000000083) [59] annotated with molecular subtypes, with 215 and 1,784 samples respectively. The breast cancer molecular subtypes are defined by expression signatures through the PAM50 subtype definition [70] and accurate prediction of basal and luminal-A subtypes and recapitulation of their corresponding expression signatures served as an important validation of our classifier.

The ovarian cancer subtype predictor (Fig. 5d) attained an overall accuracy of 84.19%(n=4) (the best performance was for mesenchymal: 98.5%) while the breast cancer subtype predictor (Fig. 5e) achieved an overall accuracy of 79.88% (n=4) (the best performance is for basal-like: 99.2%). We also compared our result against the PAM50 classifier [71] in breast cancer, and PAM50 classifier only achieved 77.35% (n=4) accuracy, because PAM50 classification is mainly based on linear correlation, while our random forest approach can also handle the nonlinear associations. Besides, our nested cross-validation approaches demonstrated that the accuracy obtained by the random Forest model is robust to changes in the hyperparameters used including the number of features used at each node (data not shown here). Our model also demonstrated a higher accuracy for identifying the basal-like subtype of breast cancer for which a diabetes drug was shown to be a potential therapy [72].

## 5 Discussion

It is widely appreciated that cancer is a disease at the scale of the entire genome, but it remains difficult to effectively translate this complexity into clinical utility, especially in CUP. An important piece of information that is relevant for clinical care in all cancer settings is knowledge of primary tissue of origin. It can, therefore, be presumed that identifying tissue of origin and perhaps even the molecular subtype of a CUP tumour is required to guide optimal treatment [73]. We have therefore used publically available datasets representing 32 cancer types from two large pan-cancer genome consortia datasets to develop a tissue of origin prediction method. We also extended beyond the tissue of origin to incorporate molecular subtyping for 11 common cancer types to provide further resolution and potential clinically relevant information. Importantly, the classifier was validated using the widely available RNA-seq method and validated on a series of archival FFPE samples replicating conditions widely experienced in diagnostic laboratories.

The performance of our classifier was similar to that reported in other studies achieving an accuracy of 75.7%. Importantly, in testing CUP-AI-Dx we report the accuracy across a balanced representation of tumour types in an independent clinical series and report the accuracy of all cases tested regardless of prediction confidence score. Although CUP-AI-Dx performs well for most cancers, some tumour types showed a drop in classification accuracy. Classification can be challenging among tumours of the same organ system such as gastrointestinal tumours and also among tumours with similar histology, such as squamous cell carcinoma. Classification inaccuracies were noted in our clinical series among pancreatic adenocarcinoma and cholangiocarcinomas which is not surprising given that in clinical practice this distinction cannot be made with certainty in many cases [74]. It is worth noting that cholangiocarcinomas were not included in the prediction model or tested in some previous methods including the DNA methylation-based test EPICUP [75], while pancreatic adenocarcinomas and cholangiocarcinomas are combined as single class pancreatobiliary for the CancerTypeID 92 gene RT-PCR test [15]. The distinction between adenocarcinomas of the ampulla, bile duct, and pancreas as well as intrahepatic cholangiocarcinoma and metastatic carcinoma are known diagnostic dilemmas where immunohistochemistry is of limited value and separation in clinical practice my be prone to interobserver discordance [74,76]. That is, we believe that caution may be required for interpretation of gene-expression classification among this group as the gold standard may be imperfect. Even incorporating orthogonol evidence such as mutation analysis with gene-expression prediction may be only partially useful in resolving some pancreatobiliary tumors [8]. A technical caveat in testing metastatic samples is also the potential for contaminating normal tissue. Such examples were evident in our clinical validation series of known metastatic tumours (Table S10), where the top prediction corresponded to biopsy site and not cancer type. In this regard consideration of the second-highest prediction can sometimes be informative.

Although our study performs well on identifying most solid cancers, it may not account for all biological differences observed among tumours or adequately represent rare cancers. The tissue of origin diagnostics, whether IHC-based or using molecular profiling, makes the fundamental assumption that metastases, including CUPs, retain features of the primary cell or tissue of origin. Although in this study we did not test CUP tumours, previous studies have shown that latent primary CUP, where a primary becomes known in time, can be predicted with similar accuracy to metastatic tumours of known origin [13,37], which suggests these tools can be diagnostically useful. However, it is also apparent that some cancers can have unusual transcriptional and epigenetic profiles. For instance, a previous study based on unsupervised analysis of TCGA data using multiple genomics platforms demonstrated tumors clustering outside of their tissue of origin, either among unrelated cancer types or clustering as a heterogeneous group independently from known tumour type clusters [77]. Anecdotal evidence of CUP tumours that do not retain features of the common tumour types in the TCGA set has also been reported [78]. These observations put fundamental limits on accuracy achievable by gene expression-based classification. Aside from challenging cases of apparent dedifferentiation or potential reprogramming, the lack of representation of many rare cancers in training is also a limitation, which in CUP-AI-Dx is the case for both neuroendocrine tumours and sarcomas. We anticipate future versions of the test to better represent such tumour types.

The path to improving the tumour type classification accuracy may be to consider including other potential features such as somatic point mutations [79] and histopathology images [80] in the model. Mutational profiling of tumours is steadily being incorporated into mainstream work-up of cancer patients and recently several tissue of origin classification methods have been developed based on DNA features alone either from panel [81] whole-exome, and whole-genome sequencing (WGS) [82]. Interestingly, the reported accuracy of these methods especially when using WGS passenger mutational profiles for the tissue of origin classification is similar to using gene-expression profiling and DNA methylation classification. Features from mutation and copy-number analysis are likely to augment both accuracy and robustness against technological and batch variation. DNA features will also help in resolving the molecular subtype of that tumour type. For example, the CIN subtype in gastric cancer is known to exhibit large structural variations that may not be captured accurately by expression data [83]. Combining orthogonal datatypes from the same patient sample is likely a rational approach to improving tumour type and molecular subtype classification and will become practical as analysis of both DNA and RNA becomes more routine for cancer patients. The three-dimensional structure of the genome is cell-type specific and therefore can add another important layer of information to improve the classification accuracy and deep neural networks like our 1D-inception models are capable of learning this latent structure as previously demonstrated [84,85].

In summary, we have demonstrated the utility of our machine learning algorithms to decode gene expression profiles and better meet the clinical challenge of identifying the primary site and the molecular subtype of multiple cancers. These predictors, including the deep learning-based predictor, will be made available as open-source software, freely available for academic non-commercial use. To make these tools available to as wide an audience as possible, we offer our models and results in a publicly available software package, which can be applied to other datasets to reproduce the results presented here.

## Appendix Supplementary materials

Table S1. Cross-validation performance metrics of primary tumour predictors Image, application 1

Table S2. External validation performance metrics of primary tumour predictors on TCGA metastatic data Image, application 2

Table S3. Cross-validation of performance metrics of random forest subtype predictors Image, application 3

Table S4. External validation performance metrics of random forest subtype predictors Image, application 4

Table S5. Contingency table and performance metrics random forest subtype predictors on pan-cancer Image, application 5

Table S6. List of 817 feature genes with Entrez Gene IDs for primary tumour classification Image, application 6

Table S7. List of features genes with gene names for 11 molecular subtype cancer classification Image, application 7

Table S8. External validation performance metrics of primary tumour predictors on clinical data Image, application 8

Table S9. Cross-validation and external validation of XGBoost performance metrics Image, application 9

Table S10. Probable cause of misclassification for samples in the clinical cohort Image, application 10

Figure S1. Additional performances of CNN models for primary tumour type prediction. Figure S2. Performance metrics for primary site predictors, 1D-CNN and Reception. Figure S3. Primary tumour type prediction performance of CNN models with a smaller feature size. Figure S4. 1D-Inception model performance with gene removal Image, application 11
