# Supplementary material for: CUP-AI-Dx: A tool for inferring cancer tissue of origin and molecular subtype using RNA gene-expression data and artificial intelligence
Source: eBioMedicine. 2020 Oct 9;61:103030. doi: 10.1016/j.ebiom.2020.103030 (PMC7553237; doi:10.1016/j.ebiom.2020.103030)
Supplement: Supplementary file 11 — Figure S1. Additional performances of CNN models for primary tumour type prediction. Figure S2. Performance metrics for primary site predictors, 1D-CNN and Reception. Figure S3. Primary tumour type prediction performance of CNN models with a smaller feature size. Figure S4. 1D-Inception model performance with gene removal [file mmc11.pptx]

## Slide 1
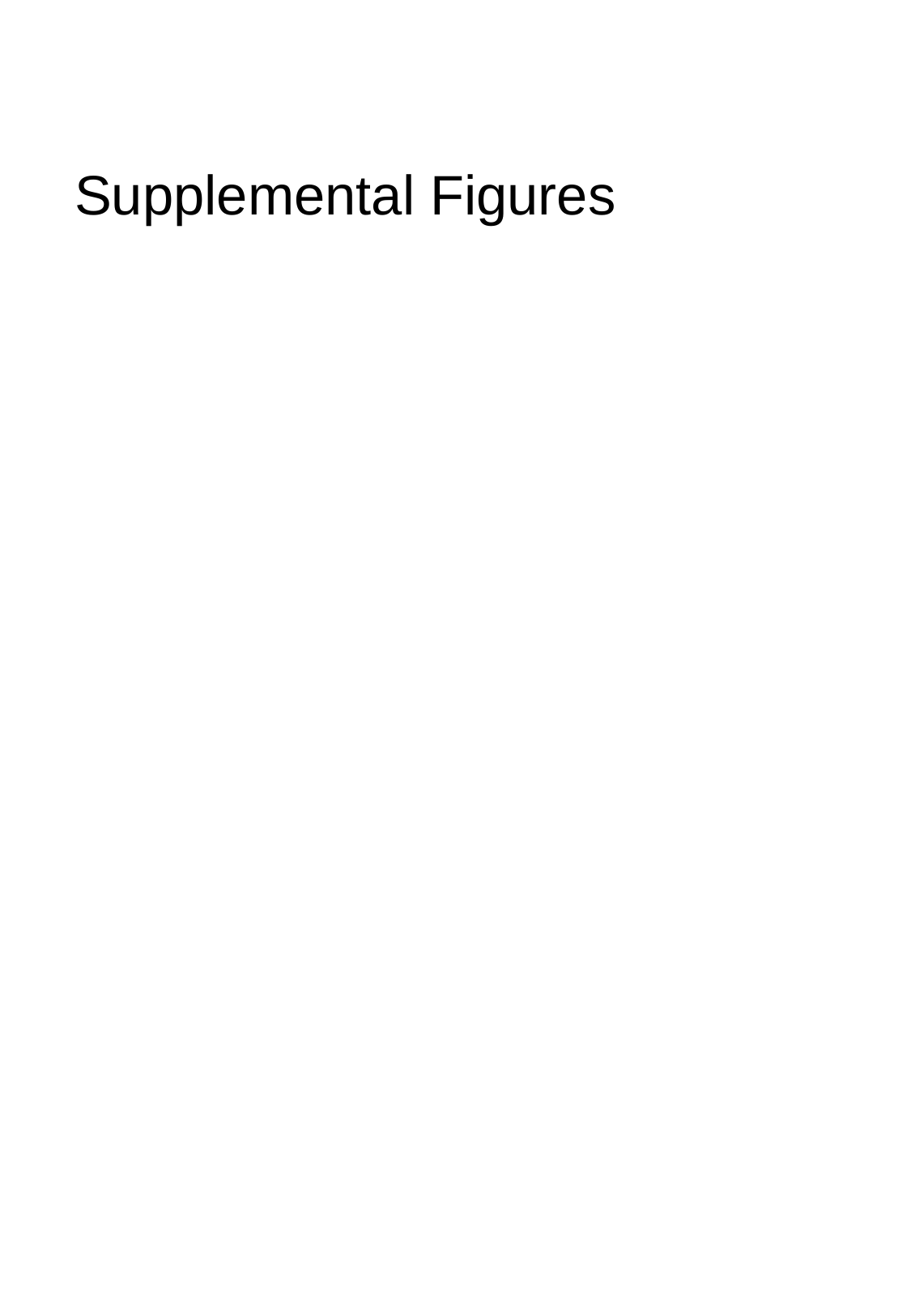

# Supplemental Figures

## Slide 2
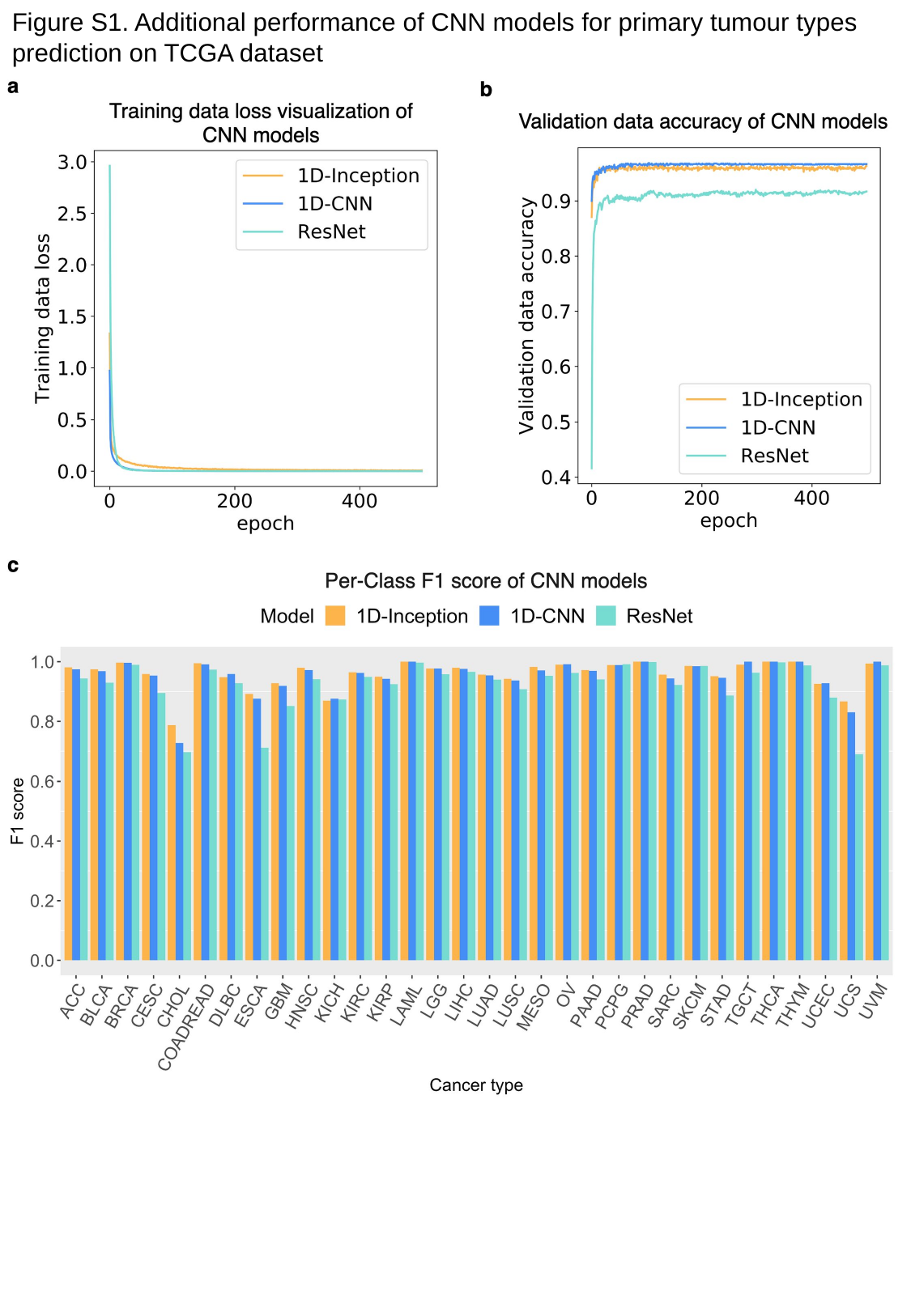

Figure S1. Additional performance of CNN models for primary tumour types prediction on TCGA dataset

## Slide 3
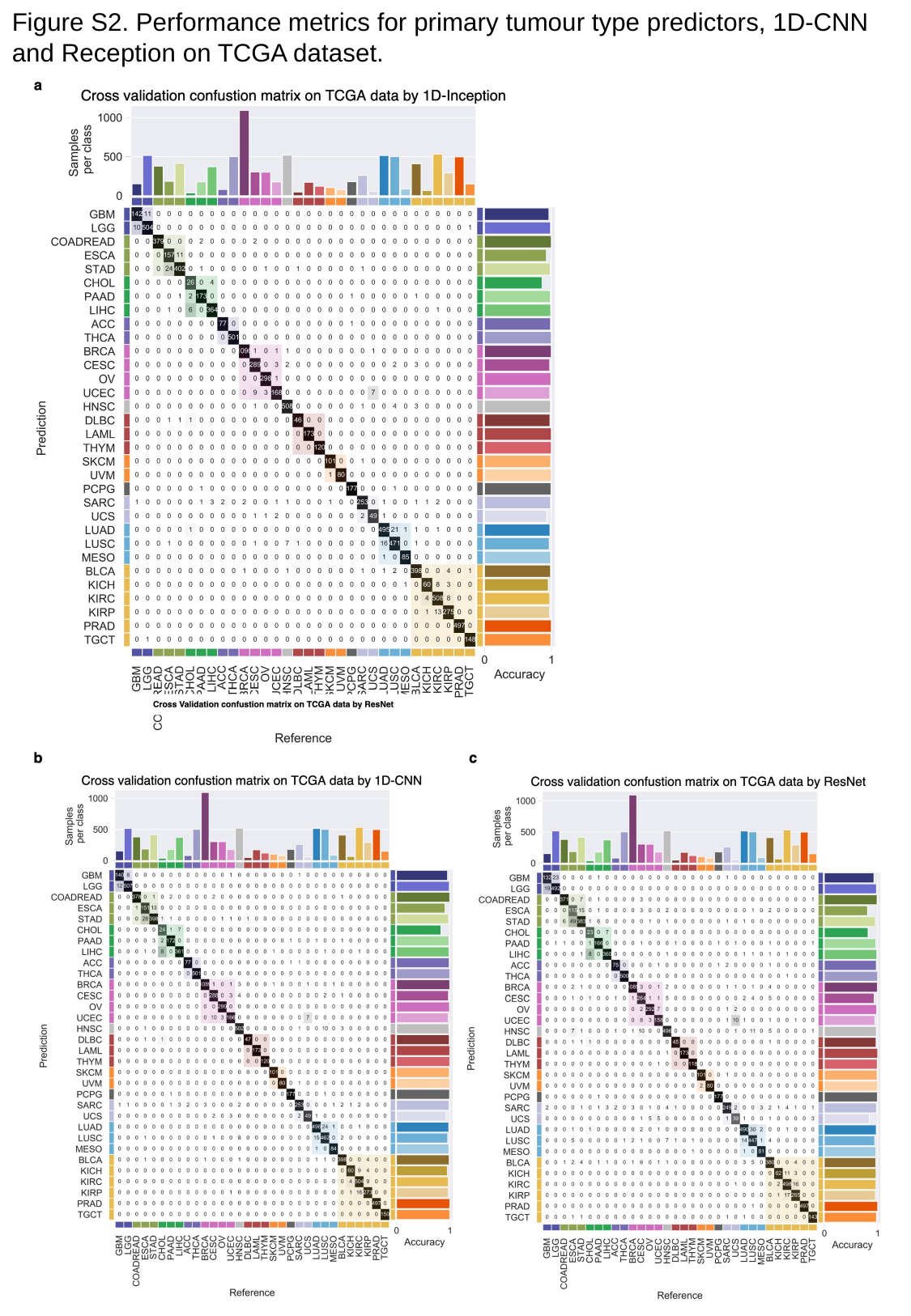

Figure S2. Performance metrics for primary tumour type predictors, 1D-CNN and Reception on TCGA dataset.

## Slide 4
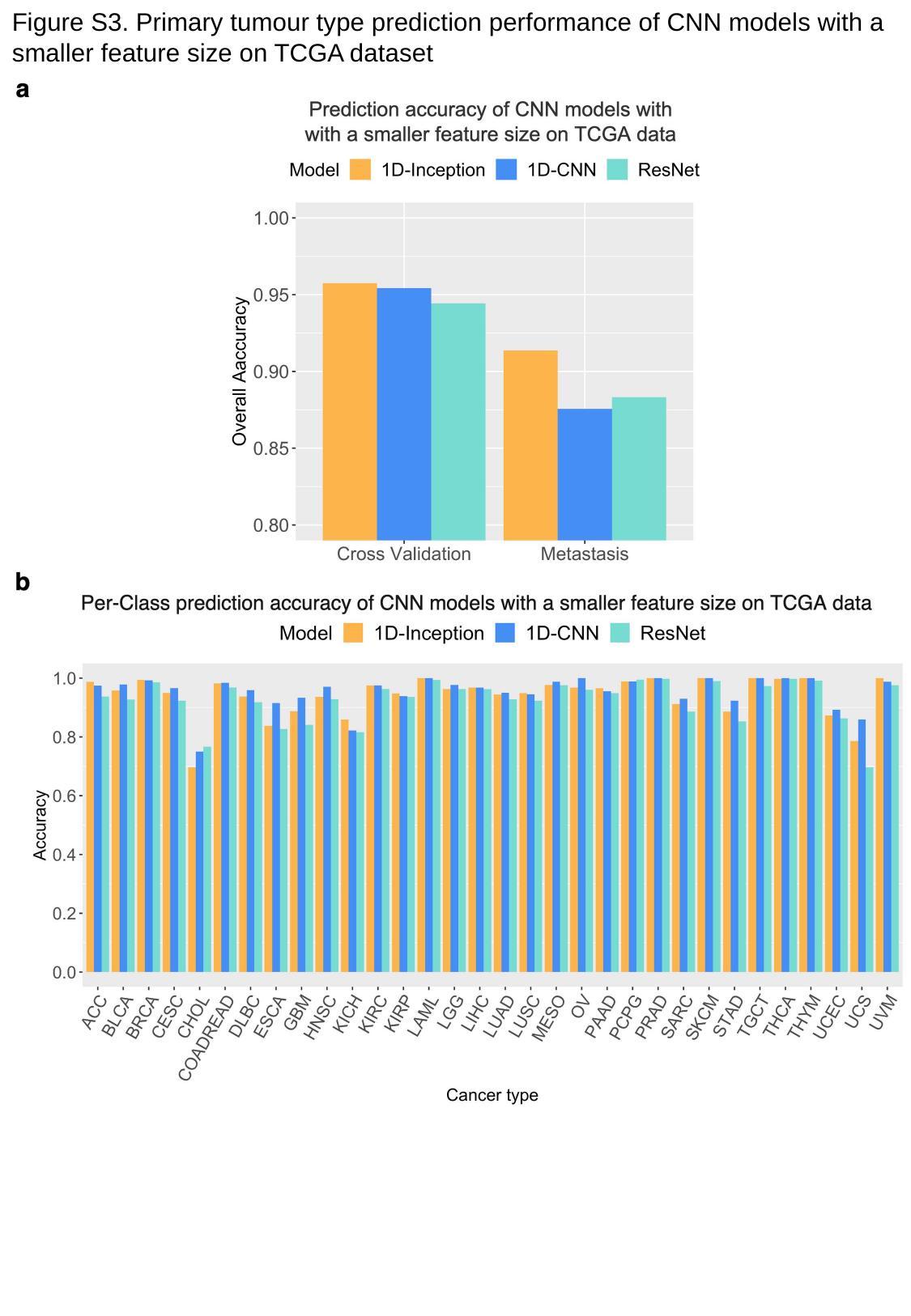

Figure S3. Primary tumour type prediction performance of CNN models with a smaller feature size on TCGA dataset

## Slide 5
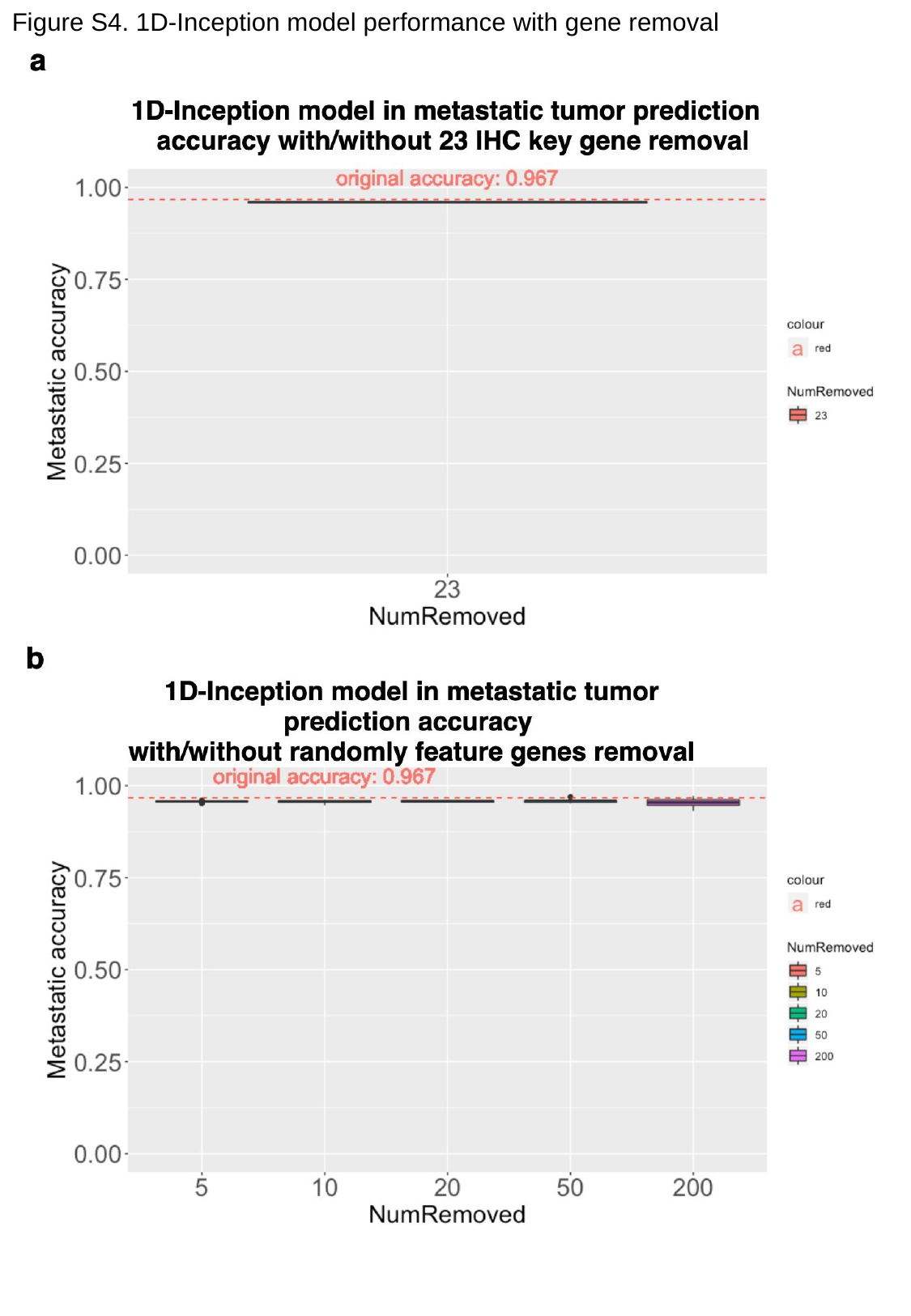

Figure S4. 1D-Inception model performance with gene removal
